# Copper doped hydroxyapatite nanocomposite thin films: synthesis, physico–chemical and biological evaluation

**DOI:** 10.1007/s10534-024-00620-2

**Published:** 2024-07-29

**Authors:** Carmen Steluta Ciobanu, Daniela Predoi, Simona Liliana Iconaru, Mihai Valentin Predoi, Liliana Ghegoiu, Nicolas Buton, Mikael Motelica-Heino

**Affiliations:** 1 National Institute of Materials Physics, Atomistilor Street, No. 405A, P.O. Box MG 07, 077125 Magurele, Romania; 2 Department of Mechanics, University Politehnica of Bucharest, BN 002, 313 Splaiul Independentei, 060042 Bucharest, Romania; 3 HORIBA Jobin Yvon S.A.S., 6-18, Rue du Canal, 91165 Longjumeau Cédex, France; 4 ISTO, UMR 7327 CNRS Université d’Orléans, 1A Rue de la Férollerie, 45071 Orléans CEDEX 2, France

**Keywords:** Hydroxyapatite, Copper, Thin films, Biocompatibility

## Abstract

Cu-doped hydroxyapatite (CuHAp) thin films were obtained using spin coating method. To make these thin films, CuHAp suspensions obtained by sol–gel method were used. The coatings obtained were thermally treated at 500 °C. After the thermal treatment, the thin films were characterized by X-ray diffraction (XRD), scanning electron microscopy (SEM). Moreover, the stability of the suspensions before being used to obtain the thin films was certified by dynamic light scattering (DLS), zeta potential methods and ultrasound measurements. In the XRD patterns, the peaks associated with hexagonal hydroxyapatite were identified in accordance with JCPDS no. 09-0432. EDS and XPS results confirmed the presence of Cu ions in the samples. Data about the morphological features and chemical composition of CuHAp thin films were obtained by performing scanning electron microscopy (SEM) measurements. Our results suggest that the CuHAp thin films surface is continuous and homogenous. The presence of the functional groups in the CuHAp thin films was confirmed by Fourier-transform infrared spectroscopy (FTIR) and Raman spectroscopy studies. Information about the surface topography of the CuHAp thin films has been obtained using atomic force microscopy (AFM). The AFM images determined that the surface topography of the CuHAp thin layer is homogenous and continuous without presenting any unevenness or fissures. The cytotoxicity of CuHAp thin films was assessed using human gingival fibroblasts (HGF-1) cells. The results of the cell viability assays demonstrated that the thin films presented good biocompatible properties towards the HGF-1 cells. Additionally, the adherence and development of HGF-1 cells on the surface of CuHAp thin films were determined using AFM. The AFM surface topographies highlighted that the CuHAp thin film’s surface favored the attachment and proliferation of HGF-1 cells on their surface.

## Introduction

In the last decade, one of the most intense studied biomaterials is represented by hydroxyapatite (HAp, Ca_10_(PO_4_)_6_(OH)_2_) (Hench 1998; Shi 2006; Unabia et al. 2018). On the other hand, HAp is an important constituent of bones, teeth and vertebrae (Nam et al. 2018; Unabia et al. 2020; Kamonwannasit et al. 2020). Bones are dynamic structures, which are responsible for support, movement, protection, mineral storage, and blood cell production. The main constituents of bones are cells (osteoblasts, osteocytes, osteoclasts), an extracellular matrix rich in collagen and hydroxyapatite. These components work together to ensure that bones are strong yet flexible, capable of growth and repair, and are able to perform their various roles in the body (Von Euw et al. 2019; Glimcher 2006). Among the elements found in the environment, copper (Cu) is particularly crucial for the body’s proper functioning. Other essential metals such as calcium (Ca), magnesium (Mg), phosphorus (P), fluoride (F), zinc (Zn), and iron (Fe) have also an important role in human and animal metabolism. In contrast, metals like lead (Pb), cadmium (Cd), and mercury (Hg) are extremely harmful and interfere with critical bodily processes. The levels of these elements in the body are influenced by both biological and environmental factors, including dietary intake, gender, age, and environmental pollution (Vidaud et al. 2012). Among the excellent biological properties of HAp we mention: bioactivity, biocompatibility, osteoconductivity and nontoxicity (Nam et al. 2018; Unabia et al. 2020; Kamonwannasit et al. 2020). A method that could lead to the improvement of HAp properties is represented by the addition/substitution of foreign ions in their structure (Bazin et al. 2021). Furthermore, previous studies have reported that by substitution with zinc, copper, silver, cerium, etc. the new obtained composite materials possess improved properties (Morais et al. 2015; Cizek et al. 2018; Predoi et al. 2019; Ghosh et al. 2019;). For example, copper ions (Cu^2+^) in small quantities are especially relevant for biomedical applications in bone regeneration, as they promote the growth of new blood vessels and enhance the survival of osteogenic cells in implants, as well as exhibit antibacterial properties (Imrie et al. 2013; Hidalgo-Robatto et al. 2018; Jacobs et al. 2020; Unabia et al. 2020). Copper plays an important role in regulation of bone growth and development of the skeleton. The element induces the formation of lysine crosslinks in collagen and elastin via lysyl oxidase activation. As a cofactor of antioxidant enzymes, it removes bone free radicals that cause the osteoclast activation (Kubiak et al. 2010). In addition, copper inhibits osteoclastic bone resorption directly (Li and Yu 2007). Altogether, copper increases bone strength and helps to maintain the optimal state of bone quality. It has been reported that in the case of mammals, copper (Cu) plays a vital role in collagen hardening, the keratinization of hair and fur. Also studies have emphasized that copper is involved in the normalization of calcium (Ca) and phosphorus (P) deposition in bones. Although, copper is essential for bone formation and mineralization, this element also serves as a cofactor for several enzymes, including lysyl oxidase, which is responsible for collagen fiber cross-linking. Disruption of this process can lead to bone weakness (Kabata-Pendias and Mukherjee 2007; Brodziak-Dopierala et al. 2009; Ciosek et al. 2023).

Moreover, the presence of traces of copper ions in the human body may improve the angiogenesis (Bazin et al. 2021). However, the use of copper in biomaterials is restricted by its potential harm to living organisms at high doses (Wang et al. 2016; Unabia et al. 2020).

In the work entitled “Sintering and biocompatibility of copper-doped hydroxyapatite bioceramics” reported by Tiphaine Bazin and coworkers, was highlighted the fact that by high temperature solid-state reaction sintering could be obtained CuHAp phase with good biocompatibility towards MC3T3-E1 cells line (Bazin et al. 2021). More than that, in the paper reported by Laura Lukaviciute et al. were presented the results of the studies conducted on the substituted calcium hydroxyapatite (copper and zinc ions) on titanium substrate obtained by low-temperature sol–gel dissolution–precipitation deposition approach (Lukaviciute et al. 2023). Their studies underlined that copper doped hydroxyapatite coatings exhibit antibacterial activity against *B. subtilis* bacterial strain (Lukaviciute et al. 2023). A new method involving two successive electrochemical reactions was used to produce copper–hydroxyapatite (Cu–HA) composite coatings on titanium with enhanced antibacterial properties (Ghosh et al. 2019). The results of the studies conducted by Rashmi Ghosh et al. showed that copper–hydroxyapatite (Cu–HA) composite coatings displayed copper concentration-dependent antibacterial activity (Ghosh et al. 2019). The results of the biological studies conducted on electrophoretic-deposited hydroxyapatite-copper coatings showed the good antibacterial activity against *E. coli* and *S. aureus* bacterial strains (Ghosh et al. 2019). Furthermore, the results of the MTT assays conducted on CuHAp coatings deposited on Ti6Al4V substrates using osteoblast-like MG63-cell line revealed that for lower copper concentration the studied coatings exhibited improved cytocompatibility (Hadidi et al. 2017).

Therefore, taking into account all these special characteristics of nanocomposite thin films based on copper-doped hydroxyapatite, it could be said that this type of biomaterials are potential candidates for the development of new metallic prostheses that could promote tissue restoration or even regeneration of severely affected bone tissue. The CuHAp thin films obtained by spin-coating presented in this paper demonstrate superior control over film thickness, better surface uniformity, and enhanced biological properties. Traditional methods like sol–gel, dip coating, or electrodeposition previously reported do not offer the same level of precision in thickness control and uniformity, and may involve more complex and expensive processes. Additionally, the biological properties introduced by copper doping are typically more pronounced in spin-coated films due to better dispersion and integration of copper ions within the hydroxyapatite matrix.

The main objective of this study is to develop by spin coating method new coatings based on copper doped hydroxyapatite (CuHAp; Ca_10-x_Cu_x_(PO_4_)_6_(OH)_2_, x_Cu_ = 0.02) on Si substrate. In order to achieve this objective, the copper doped hydroxyapatite was obtained by sol–gel method. The physico-chemical features of the new CuHAp thin films were analyzed by performing X-Ray diffraction (XRD), X-ray photoelectron spectroscopy (XPS), energy dispersive X-ray spectroscopy (EDS), Fourier-transform infrared spectroscopy (FTIR) and Raman spectroscopy studies. Also, data about the CuHAp thin films surface morphology was obtained by scanning electron microscopy (SEM) and atomic force microscopy (AFM) studies. The in vitro biocompatibility of CuHAp thin films was studied by MTT assay using human gingival fibroblasts (HGF-1) cells line.

## Materials and methods

### Synthesis of copper doped hydroxyapatite

For the development of copper doped hydroxyapatite (CuHAp; Ca_10 − x_Cu_x_(PO_4_)_6_(OH)_2_, x_Cu_ = 0.02) by an adapted sol–gel method, were used as precursor the next reagents: calcium nitrate (Ca(NO_3_)_2_·4H_2_O, ≥ 99.0%, Sigma Aldrich, St. Louis, MO, USA), copper nitrate (Cu(NO_3_)_2_ · 3 H_2_O, 94–104%, Sigma Aldrich, St. Louis, MO, USA), triethanolamine (C_6_H_15_NO_3_, Sigma-Aldrich; ≥ 99.0% (GC) purity), and ammonium hydrogen phosphate ((NH_4_)_2_HPO_4_, ≥ 99.0%, Sigma Aldrich, St. Louis, MO, USA). In the synthesis process were also used bi-distiled water and absolute ethanol (C_2_H_5_OH, Sigma Aldrich, St. Louis, MO, USA). During the synthesis the value of [Ca + Cu]/P ratio, was set at 1.67. The obtaining of CuHap suspension was made in agreement with the procedure described in detail in our previous paper (Predoi et al. 2019). Briefly, specific quantity of calcium precursor (~ 23 g) was dissolved in 50 ml of ethanol. Then, in the Ca-solution was slowly added triethanolamine (0.013 mol). Also, the stoichiometric quantity of Cu (~ 0.05 g) and P (~ 8 g) precursors were well dissolved in ethanol (50 ml). Then the solution that contain Ca was slowly incorporated in the solution that contains Cu and P. Next, the obtained mixture was stirred at 100 °C for 12 h. The next step was represented by the deposition of CuHAp on the Si substrate (7 mm × 7 mm) by spin-coating. For this purpose, a Si wafer (Siegert Wafer GmbH, Aachen, Germany) served as substrate. The coating was achieved by using 0.5 ml of CuHAp. The CuHAp was deposited onto the polished side of the Si substrate (previously washed with acetone and bi-distilled water) using a syringe. The parameters used for coatings deposition were: spin time: 90 s and speed: 2000 rpm. The deposition process was repeated 35 times and after each deposition, the sample was dried for 15 min at 100 °C in air. Finaly, the CuHAp thin films were annealed for 2 h in air at 500 °C.

### Characterization of copper doped hydroxyapatite

A X-ray diffractometer Bruker D8 Advance (Bruker, Karlsruhe, Germany) using a Cu Kα radiation (λ = 1.5418 Å) was used for the X-ray diffraction studies (XRD). The XRD experimental data were collected between 20° and 60° (2θ) range, with a step size of 0.02°.

For the X-ray photoelectron spectroscopy (XPS) studies was used a Multimethod SPECS surface analysis system (SPECS GmbH, Berlin, Germany) operating with an Al Kα monochromatic radiation (1486.6 eV). For this study was used the experimental conditions reported in the previous studies (Iconaru et al. 2021). The XPS data were analyzed using Spectral Data Processor v. 2.3 (SDP) software.

The presence of functional groups in the structure of CuHAp thin films was analyzed by Fourier-transform infrared spectroscopy (FTIR) studies. The FTIR studies were made with the aid of a Perkin Elmer SP-100 spectrometer (Waltham, MS, USA), and the experimental data were collected between 450 and 4000 cm^−1^ spectral domain.

Complementary information about the molecular structure of CuHAp thin films were obtained with the aid of Raman spectroscopy. For this purpose, was used a Horiba Jobin Yvon LABRAM HR Evolution spectrometer. Raman spectroscopy studies were performed at room temperature, using a HeNe laser (633 nm). Raman spectra were collected between 400 and 1200 cm^−1^ spectral range.

The surface morphology of CuHAp thin films was studied with the aid of a FEI Quanta Inspect F scanning electron microscope (FEI Company, Hillsboro, Oregon, United States). The microscope was also equipped with an energy-dispersive X-ray (EDS) attachment in order to evaluate the chemical composition of CuHAp thin films.

Data regarding the morphological features of CuHAp thin films were obtained by performing scanning electron microscopy measurements. By performing Atomic Force Microscopy (AFM) measurements were obtained additional information regarding the surface topography of CuHAp thin films. For this purpose, a NT-MDT NTEGRA Probe Nano Laboratory (NT-MDT, Moscow, Russia) instrument was used. The studies were done using semi-contact mode. The AFM images were recorded on surface areas of 5 × 5 µm^2^ using a silicon NT-MDT NSG01 cantilever (NT-MDT, Moscow, Russia) with a 35 nm gold layer. The roughness of the CuHAp thin films’s surface was also evaluated with the aid of roughness parameter R_RMS_. The Gwyddion software (2.59 version, Department of Nanometrology, Czech Metrology Institute, Brno, Czech Republic) was used to analyze the AFM experimental data (Gwyddion software 2023). For the Dynamic light scattering (DLS) and ζ-potential studies performed on CuHAp suspensions a SZ-100 Nanoparticle Analyzer (Horiba, Ltd., Kyoto, Japan) was used. An adapted procedure was used for the DLS and ζ-potential measurements. These procedure was described in detail in our previous work (Predoi et al. 2019). Also, the ultrasonic tests were effectuated using the setup and the parameters indicated in our previous paper (Predoi et al. 2019).

### Biological evaluation of copper doped hydroxyapatite

The cytotoxicity of CuHAp thin films was assessed with the aid of human gingival fibroblasts (HGF-1) cells as previously described in (Ciobanu et al. 2022). For this purpose, the cells were grown in Dulbecco’s Modified Eagle’s Medium enriched with heat-inactivated fetal bovine at 37 °C in an atmosphere with 5% CO_2_. HGF-1 cells were incubated for 24, 48 and 72 h with the CuHAp thin films and their cytotoxicity was assessed by determining the cell viability using the reduction assay MTT [3-(4,5dimethylthiazolyl)-2,5-diphenyltetrazolium bromide]. For this purpose, atthe end of the incubation period, the culture medium was removed, washed using phosphate buffer saline (PBS) and the cells were incubated with 1 mg/ml MTT for 3 h at a temperature of 37 °C. The purple formazan crystals formed in the viable cells were dissolved with 2-propanol (Sigma-Aldrich, USA) and the absorbance of the medium was measured at 595 nm with the aid of a TECAN spectrophotometer. The cell viability of the HGF-1 was quantified from the optical density obtained from the absorbance measurements. The percentage of the HGF-1 viable cells were quantified by rapport to the control sample, which was considered to have a viability of 100%. Complementary data regarding the attachment and proliferation of the cells on the CuHAp thin film’s surface was attained by AFM investigation. In order to achieve this, the cells were fixed on the CuHAp thin films surface using a 70% ethanol solution and then AFM topographies of the thin films were acquired on a surface area of 40 µm × 40 µm at room temperature using the NT-MDT NTEGRA Probe Nano Laboratory instrument.

## Results

**Fig. 1:**
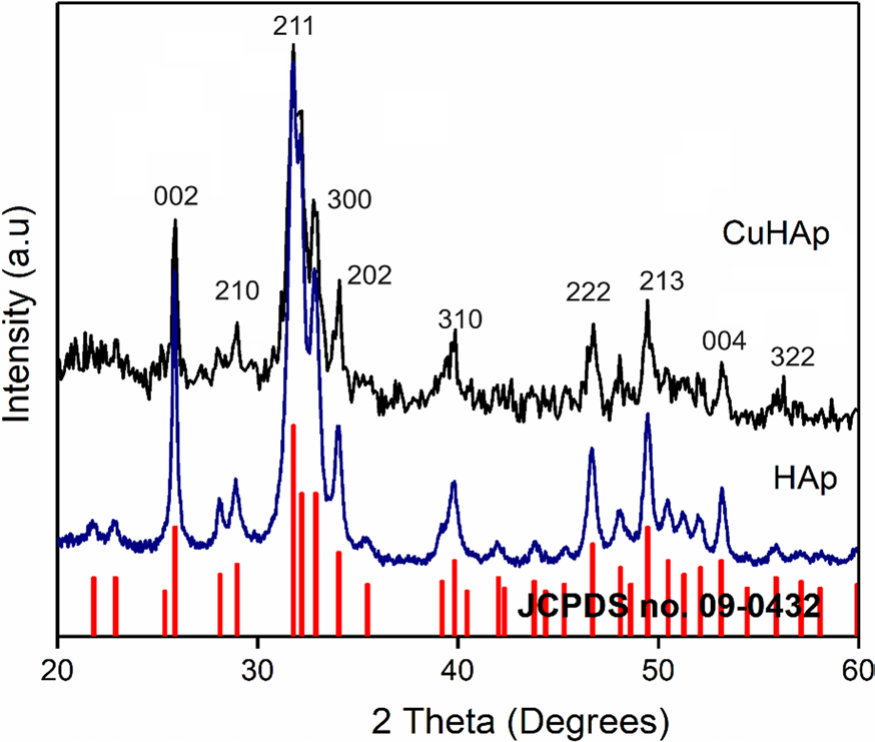
The XRD pattern of the HAp (dark blue) and CuHAp thin films (black) and JCPDS no. 09-0432 (red). (Color figure online)

The presence of hydroxyapatite in the deposited CuHAp thin films was highlighted by the results of the X-ray diffraction analysis (Fig. 1). The XRD pattern of the CuHAp thin film shows maxima associated with pure hexagonal hydroxyapatite in accordance with JCPDS no. 09-0432. The diffraction peaks associated with the family of (002), (210), (211), (300), (202), (310), (222), (213), (004) and (322) planes of the hexagonal HAp structure were identified. Peaks associated with impurities were not observed. The results of the XRD studies show that the obtained thin films present a single phase associated with HAp.

The results of the XRD studies show that the obtained thin films present a single phase associated with HAp. The crystallite sizes for the HAp (x_Cu_ = 0) and CuHAp (x_Cu_ = 0.02) samples were calculated using Scherrer’s equation (Mansour et al. 2017; Elashmawi and Manazea 2019; Ismail et al. 2019). The crystallite size decreased from 19.97 nm (HAp) to 15.53 nm for the CuHAp sample. The variation in crystallite size is due to the influence of Cu^2+^ ions on the lattice parameters of the HAp. Moreover, as a result of Cu^2+^ doping, a change in lattice parameters occurs (Table 1).

**Table 1:** Structural parameters of HAp and CuHAp

Sample	Lattice parameters		Volume (Å)^3^	D (nm)
	a (Å)	c (Å)		
HAp	9.409	6.879	527.39	19.97
CuHAp	9.398	6.871	525.54	15.53

A decrease in the lattice parameters and a contraction of the cell volume in the case of the CuHAp sample is observed as a result of the replacement of Ca (II) sites with copper. This behavior is due to the fact that the ionic radius of Cu^2+^ (r_i_ = 0.73A) is smaller than that of Ca^2+^ (r_i_ = 0.99A). This behavior is in agreement with previously presented studies (Mariappan et al. 2022).

**Fig. 2:**
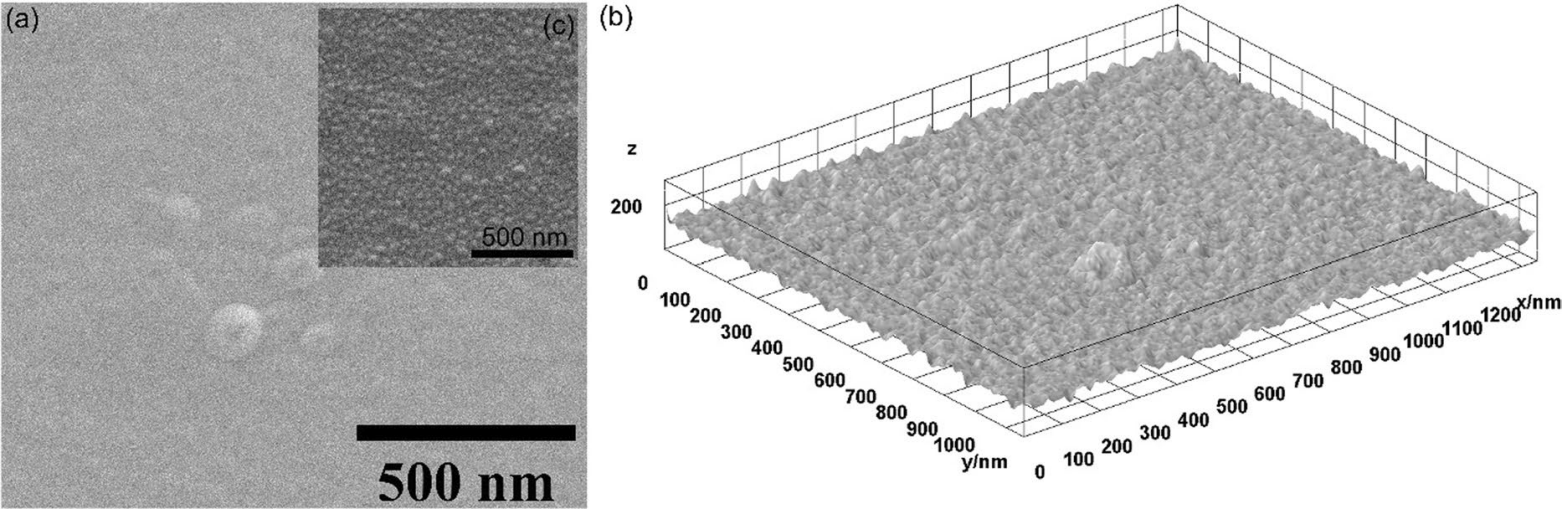
2D (**a**) and 3D (**b**) and high magnification SEM inset (**c**) representation of SEM images obtained for CuHAp thin films

In Fig. 2 are revealed both the 2D and the 3D representation of the SEM images obtained on the CuHAp thin films. In the Fig. 2 could be observed the absence of cracks and fissures on the CuHAp thin films surface. Other features that were revealed by the SEM images is represented by the fact that the CuHAp thin films surface morphology is continuous and homogenous. Moreover, no other surface defects could be noticed in the obtained 2D and 3D SEM image (Fig. 2). Furthermore, an inset of SEM at high magnification is presented in Fig. 2c. The SEM image depicts that the surface morphology of the CuHAp thin films consist of spherical nanometric particles.

**Fig. 3:**
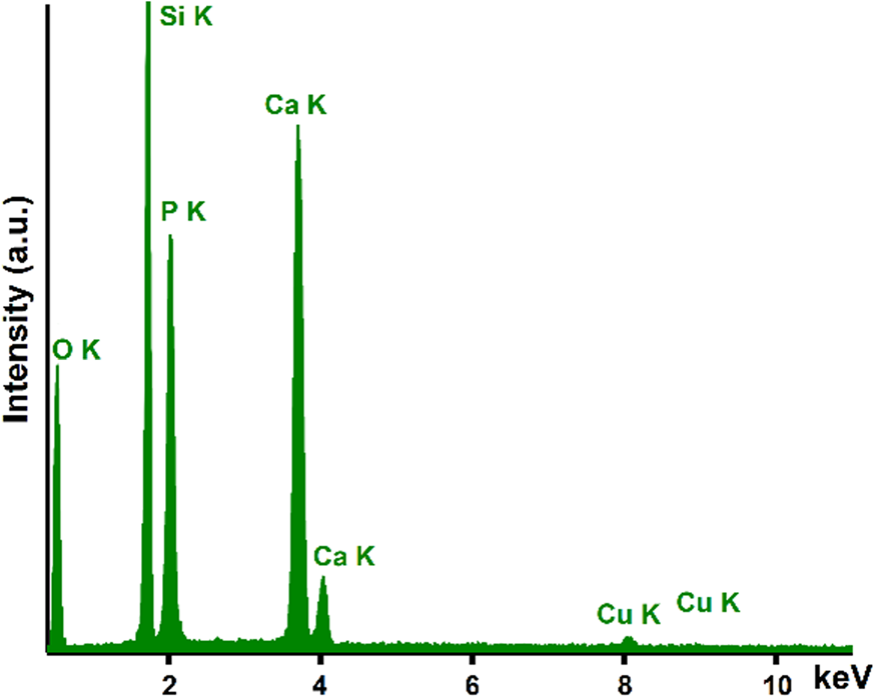
EDS spectra characteristic for CuHAp thin films

In Fig. 3 the EDS spectra that was obtained for the CuHAp thin films deposited on Si substrate is presented. Our EDS results, revealed that the obtained thin films are pure (due to the fact that in the EDS spectra cannot be observed the presence of maxima that could be attributed to contaminants). Furthermore, the EDS spectra revealed the presence of Calcium (Ca), Copper (Cu), Oxygen (O) and Phosphorus (P) in the CuHAp thin films. All this chemical elements belong to the structure of Ca_10−x_Cu_x_(PO_4_)_6_(OH)_2_. The presence of Si line in the obtained EDS spectra is due to the CuHAp thin films substrate. The results of EDS semi-quantitative analysis revealed that next wt% values were obtained for each constituent element: Calcium (34.74 wt%), Copper (0.05 wt%), Oxygen (49.1 wt%) and Phosphorus (16.11 wt%).

**Fig. 4:**
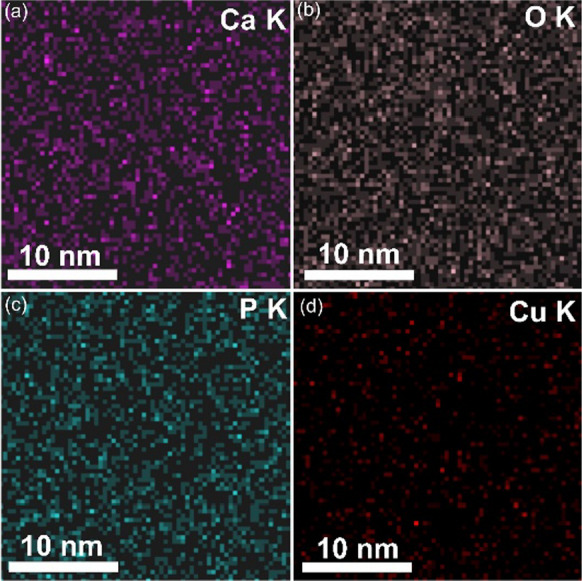
Elemental EDS cartographies obtained for CuHAp thin films : Ca (**a**), O (**b**), P (**c**) and Cu (**d**)

Also, for the CuHAp thin films were recorded the elemental EDS cartographies and they are presented in Fig. 4. The elemental EDS cartographies revealed the well and homogeneous repartition of Ca, Cu, P and O in the CuHAp thin films.

**Fig. 5:**
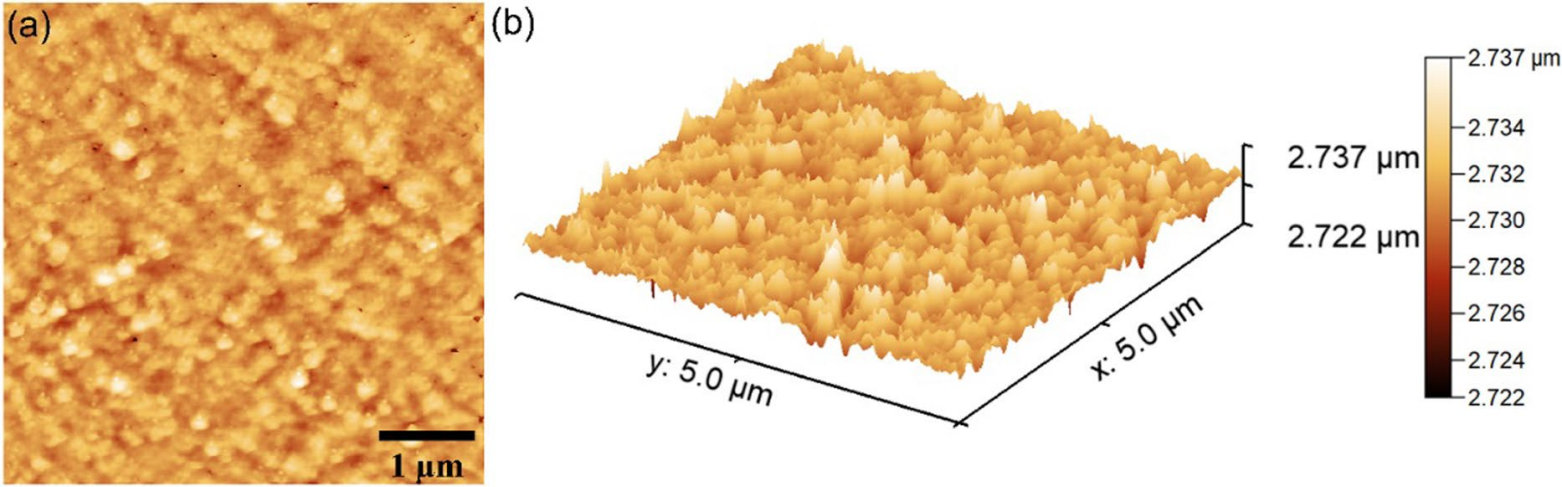
2D AFM surface topography (**a**) and 3D representation (**b**) of CuHAp thin films

Information about the surface topography of the CuHAp thin films was acquired using AFM studies. The results of the 2D AFM surface topography as well as its 3D representation is portrayed in Fig. 5a, b.

The data showed by the 2D AFM images obtained on the surface area of 5 × 5 µm^2^ revealed that the surface of the CuHAp thin films is homogenous and continuous without presenting any signs of unevenness, cracks or any other discontinuities. Furthermore, the 3D representation of the AFM topography highlighted that the surface topography of the CuHAp thin films does not present any significant irregularities and that the CuHAp exhibit the appearance of a uniformly and continuous deposited layer. In addition, the roughness parameter, R_RMS_ was determined for the entire acquired surface. The calculated R_RMS_, roughness parameter had a value of 1.33 ± 0.25 nm. The data suggested that the surface topography present a homogeneous distribution with a minimum roughness.

**Fig. 6:**
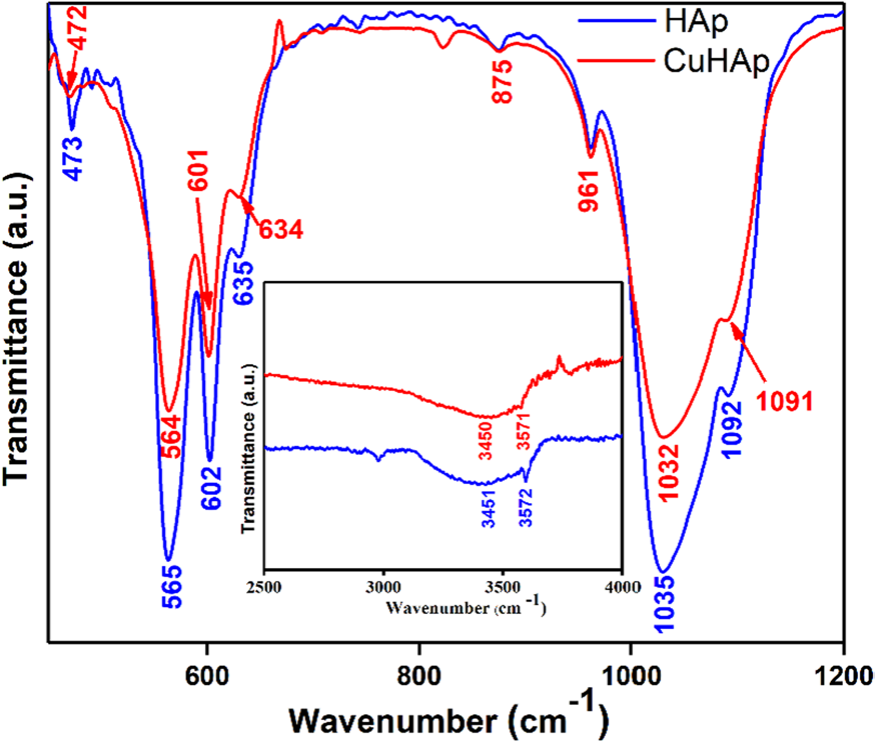
The FTIR spectra, in transmittance mode, obtained for the HAp and CuHAp thin films

The results of the FTIR studies conducted on the HAp and CuHAp thin films in order to evaluate the presence of the functional groups, are revealed in Fig. 6. The presented spectra were recorded in transmittance mode between 450 and 4000 cm^−1^. In this spectral domain could be noticed the vibrational bands that suggest the presence of phosphate and hydroxyl groups in the HAp and CuHAp sample. Also, could be observed the presence of the of vibrational bands that are specific to adsorbed water molecules, fact which suggests that the analyzed samples are hydrated.

Our FTIR results clearly indicate the presence of vibrational modes corresponding to phosphate and hydroxyl groups. For the CuHAp sample the presence of a vibration band attributed to the OH^−^ vibration could be observed at around 634 cm^−1^ and ~ 3571 cm^−1^ (Barinov et al. 2006; Qian et al. 2008; Ciobanu et al. 2013). Moreover, the vibrational band observed at ~ 3450 cm^−1^ corresponds to the adsorbed water molecules (Qian et al. 2008; Ciobanu et al. 2013). On the other hand, intense vibrational bands characteristics of PO_4_^3−^ groups from HAp structure are clearly observed in Fig. 6 at approximately 472 cm^−1^, 564 cm^−1^, 601 cm^−1^, 961 cm^−1^, and between 1032 and 1091 cm^−1^ (Bai et al. 2010; Ciobanu et al. 2013). Thus, the vibrational band centered at ~ 472 cm^−1^ is attributed to ν_2_ of PO_4_^3−^ groups. The bands centered at about 564 cm^−1^ and ~ 601 cm^−1^ usually are attributed to ν_4_ of PO_4_^3−^ groups (Bai et al. 2010; Ciobanu et al. 2013). The band observed at ~ 961 cm^−1^ is characteristic to ν_1_ of PO_4_^3−^ groups. Moreover, the vibrations bands observed between 1032 and 1091 cm^−1^, are specific to ν_3_ of PO_4_^3−^ groups from hydroxyapatite structure (Bai et al. 2010; Ciobanu et al. 2013). Finaly, the vibration band noticed at around 875 cm^−1^ in the FTIR spectra arise due to the presence of HPO_4_^2−^ ions (Mortier et al. 1989; Ciobanu et al. 2013) in the CuHAp sample. The FTIR results reveal that for the HAp thin films, the main vibrational bands associated to the PO_4_^3−^ groups (ν_1_, ν_2_, ν_3_, ν_4_), HPO_4_^2−^ groups, and OH^−^ groups, are slightly shifted comparative with the position of maxima of the CuHAp thin films. On the other hand, could be observed in the obtained FTIR spectra’s that the substitution of calcium ions (from HAp structure) with copper ions induce an intensity decrease of the CuHAp—FTIR maxima. Furthermore, the FTIR results suggest that doping hydroxyapatite with copper does not lead to the formation of additional phases. All these results are in concordance with the studies previously reported in the literature (Iconaru et al. 2014; Uysal et al. 2021).

**Fig. 7:**
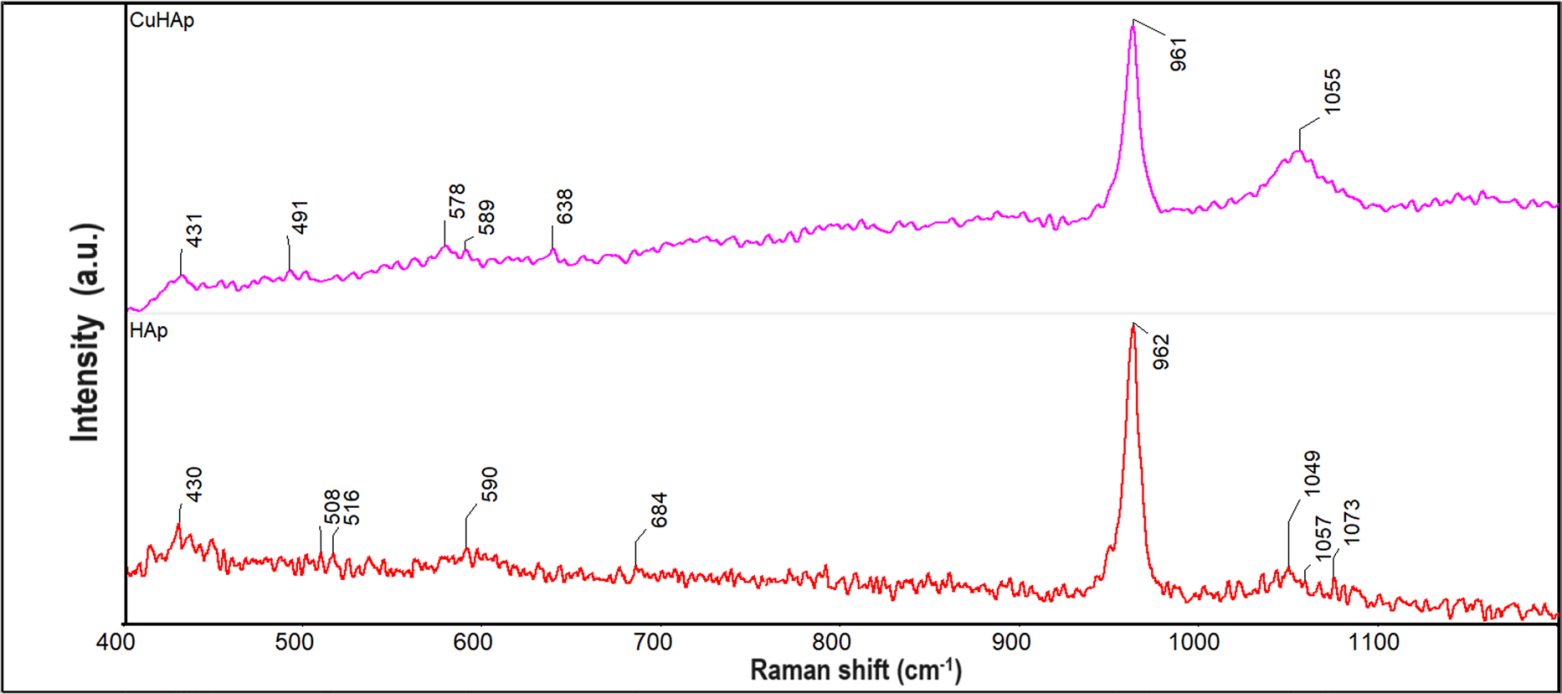
Raman spectra of HAp and CuHAp thin films

Complementary information’s with the one obtained by FTIR, about the presence of the vibrational bands that are characteristics to the hydroxyapatite structure in the HAp and CuHAp sample, were obtained by Raman spectroscopy studies. In Fig. 7 are presented the Raman spectra’s characteristic for the HAp and CuHAp thin films. The Raman spectra were recorded between 400 and 1200 cm^−1^.

The most intense vibration band observed in Fig. 7 is the one centered at ~ 961 cm^−1^ and could be attributed to the symmetric stretching of P–O bonds (ν_1_ (PO_4_^3−^)) (Ciobanu et al. 2013). The vibrational bands observed at ~ 431 cm^−1^ (*ν*_2_) and ~ 490 cm^−1^ (*ν*_2_) are specific to the O–P–O bending modes from HAp (Ulian et al. 2013;). Furthermore, the ~ 578 cm^−1^ band appear due to the *ν*_4_ vibration of (PO_4_^3−^) groups (Ciobanu et al. 2013). The OH^−^ groups vibrational band could be observed at around ~ 638 cm^−1^ in the Raman spectrum. Finally, the large vibration band centered at ~ 1050 cm^−1^ (ν_3_), could be assigned to the asymmetric ν_3_ (P–O) stretching (Ciobanu et al. 2013). No other intense bands could be detected in Fig. 7 and therefore we could say that our samples are pure. Also, these data are in good agreement with the ones previously reported (Ciobanu et al. 2013). As can be seen in the Raman spectra (Fig. 7) similar values of the maxima are obtained for the HAp sample as the one obtained for the CuHAp. The results of the Raman spectroscopy studies confirm the presence of the HAp (indicated by the maxima characteristic for ν_1_, *ν*_2_, ν_3_ of (PO_4_^3−^) groups from HAp structure) in the studied samples. Furthermore, could be observed that for the CuHAp sample the maxima are slightly shifted and broadened. These results are in good agreement with the one previously reported by (Unabia et al. 2019).

**Fig. 8:**
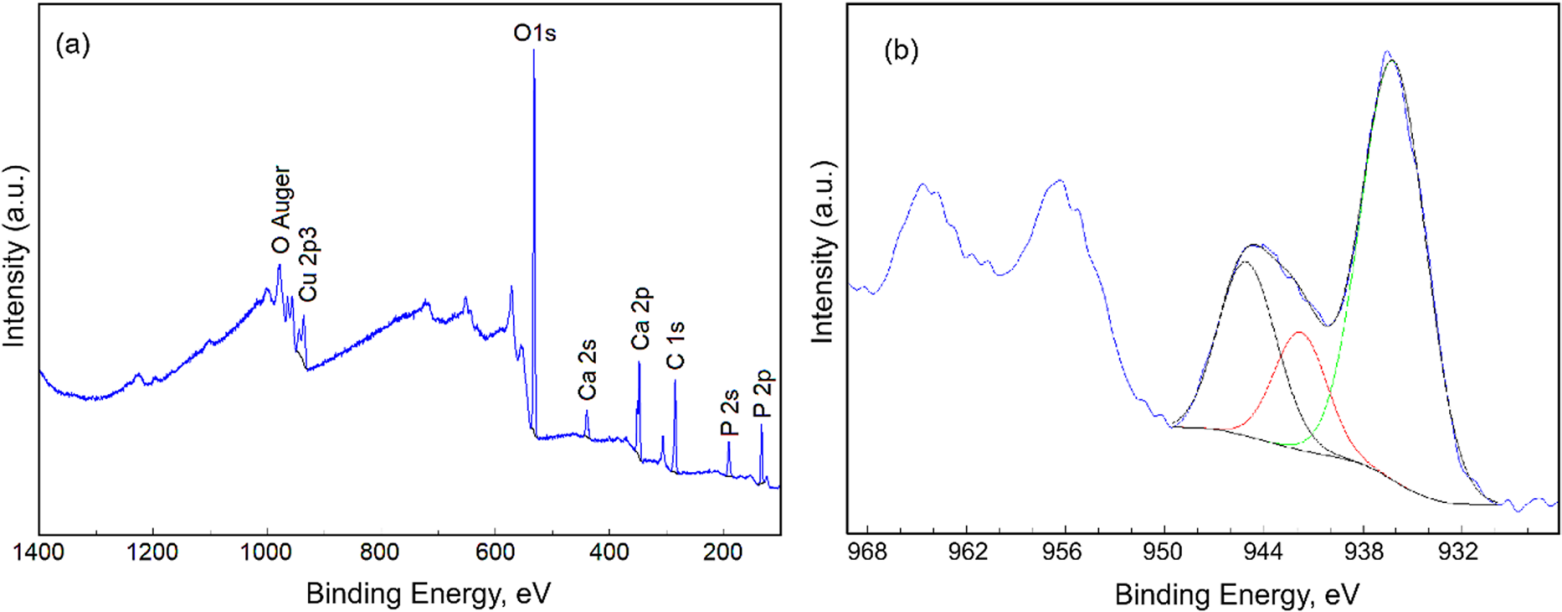
XPS survey spectra (**a**) and high resolution spectra (**b**) of the CuHAp thin films

For the surface characterization of the CuHAp thin films we used X-ray photoelectron spectroscopy (XPS) which is one of the most important techniques for surface characterization. The XPS survey spectra of the CuHAp thin films are shown in Fig. 8. The qualitative analysis highlighted both the presence of the constituent elements of HAp (Ca, P, O) and the presence of Cu. The results are in agreement with those obtained from EDS investigations and confirm the presence of copper in the analyzed samples.

Figure 8b shows the high resolution XPS spectra of Cu from CuHAp sample. The three maxima observed as a result of the deconvolution were observed at the binding energy of 936.16 eV, 941.79 eV and 945.05 eV. The peak observed at 936.16 eV was associated with Cu2p3/2. The peaks observed at 941.79 eV and 945.05 eV are shake-up peaks that indicate the presence of Cu^2+^ (Schon 1973; Zhang et al. 2006; Chusuei et al. 1999). The binding energy of Cu 2p1/2 at 952.3 eV which is characteristic of Cu ^1+^ (Kumar et al. 2017) was not observed. Moreover, the peak at 933.3 eV that indicate the existence of CuO were not observed (Chen et al. 2011).

The creation of stable nanoparticle suspensions plays an important role both in research and from an applications point of view. Stable suspensions have a wide applicability in water desalination, cosmetics, pharmacy, nanomedicine, etc. In making coatings with antimicrobial properties through the spin coating technique, the stability of nanoparticle suspensions is very important. As a result, the stability of CuHAp suspensions was evaluated both by the classic dynamic light scattering (DLS) and zeta potential methods and by ultrasound measurements. Thus, the DLS studies highlighted the fact that the suspensions are monodisperse and have a hydrodynamic diameter of nanoparticles in suspension of 35 nm. The analysis of the zeta potential showed that the suspension of CuHAp used in the production of thin films with biocompatible properties presented a negative potential with a value of approximately − 30.9 mV. Moreover, the ultrasound studies allowed us to analyze the concentrated suspension obtained without the need for successive dilutions (for the DLS and zeta potential studies the obtained suspensions were diluted 10 times in double distilled water). For the ultrasound studies, double distilled water was chosen as the stable reference suspension. The calculated average stability parameter $S = \frac{dA}{{Adt}}$ was equal to 4.9e-06 (1/s), highlighting a very good stability of the CuHAp nanoparticle suspension. The results regarding the stability of CuHAp suspensions by different analysis methods led to highlight their good stability.

**Fig. 9:**
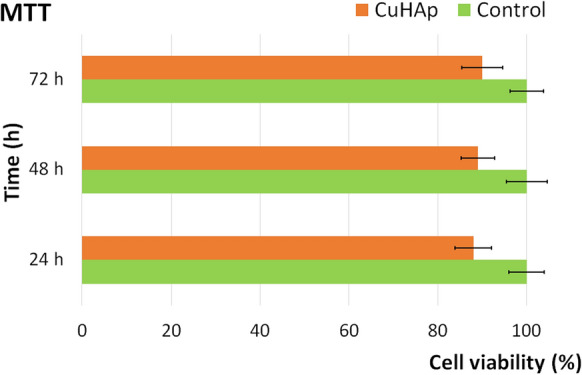
The graphical representation of the cell viability of HGF-1 cells incubated with CuHAp thin films for 24, 48 and 72 h. Data is represented as mean ± standard deviation (SD) and quantified as percentages of control (100% viability). The statistical analysis was performed using ANOVA single-factor test and p ≤ 0.05 was accepted as statistically significant

The biocompatibility of the CuHAp thin films was investigated with the aid of HGF-1 cell line. The cytotoxicity of the CuHAp thin films was studied by determining the cell viability of the HGF-1 cells using the reduction MTT assay after being incubated with the CuHAp thin films for 24, 48 and 72 h. The findings of the MTT assay were graphically represented in Fig. 9. The results were represented as mean ± standard deviation (SD) of three experiments and the values were quantified as percentages of control (100% viability). The statistical analysis was performed using ANOVA single-factor test and p ≤ 0.05 was accepted as statistically significant. The data showed that the HGF-1 cell viability was not significantly modified compared to the control cell culture after 24, 48 and 72 h of incubation with the CuHAp thin films, which indicates a good biocompatibility of the thin films. The values of the cell viability determined by the MTT assay depicted graphically in Fig. 9, showed that the cell viability values were kept above 88%in the first 24 h of incubation. Furthermore, the data also depicted that the cell viability increased with the incubation time going up to 90% and 92% respectively, after 48 h and 72 h of incubation. These findings are in accord with other reported data regarding the biocompatibility properties of hydroxyapatite and hydroxyapatite doped with various ions (ISO standard 2009; Letelier et al. 2010; Sahithi et al. 2010; Li et al. 2010, 2019; Rodríguez-Valencia et al. 2013; Geng et al. 2016; Marques et al. 2017; Gritsch et al. 2018; Hidalgo-Robatto et al. 2018; Unabia et al. 2020; Bazin et al. 2021; Ciobanu et al. 2022; Tuntun et al. 2023;). More than that, the cell viability obtained for the HGF-1 cells after being incubated with CuHAp thin films was above 88%, which is above the cytotoxicity threshold fixed at 70% by the ISO standard 10,993–5 (ISO standard 2009). The data suggested that CuHAp thin films are within the non-cytotoxic range and exhibit a good biocompatible character with the HGF-1 cells. Therefore, CuHAp thin films could be taken into consideration for the development of superior implant materials with a good cytotoxicity level.

**Fig. 10:**
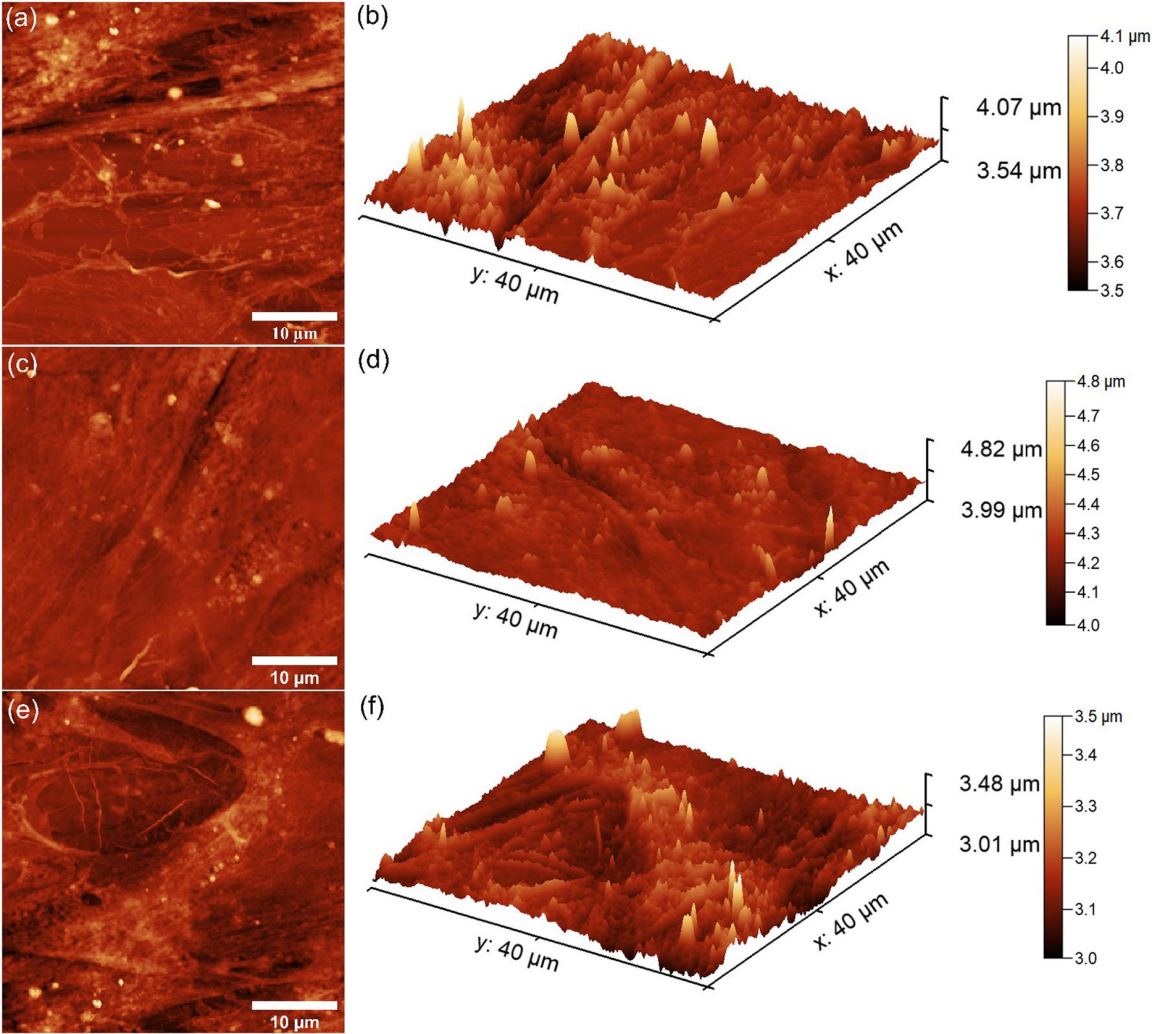
2D AFM topography of HGF-1 cells after 24, 48 and 72 h of incubation with CuHAp thin films (**a**, **c**, **e**) and their 3D representation (**b**, **d**, **f**)

Additional information about the adherence and development of HGF-1 cells on the surface of CuHAp thin films was gathered by AFM studies. The 2D AFM topographies of the thin films’ surfaces after being incubated for 24, 48 and 72 h with HGF-1 cells were acquired at room temperature and in normal atmospheric conditions on a surface of 40 × 40 µm^2^. The AFM topographies of the HGF-1 adhesion onto the surfaces of the CuHAp thin films are depicted in Fig. 10a–f.

The 2D AFM topographies showed that the HGF-1 cells adhered on the entire surface of the CuHAp thin films. Furthermore, the 2D AFM images highlighted that the adhered cells present the typical patterns of the cellular morphology of normal healthy gingival fibroblast with flattened and elongated shapes. More than that, the images depicted that the cells colonized the surface growing in confluent layers. Also, both the 2D AFM topography as well as their 3D representation of the CuHAp surface thin films suggested that after 72 h of incubation the thin films were completely covered and also that an extracellular matrix with a complex network of filopodia was observed on the surface of CuHAp thin films for all the tested incubation times (Al-Sabek et al. 2005; Rodríguez-Valencia et al. 2013; Geng et al. 2016; Warowicka et al. 2016; Mukaddam et al. 2021). The presence of filopodia indicates the good biocompatibility of the surface due to the fact that filopodia enable the cells to make contact both with the substrate and with the neighboring cells (Geng et al. 2016). Usually, the presence of filopodia and lamellipodia are the evidence of cell adhesion and spreading, indicating that the materials do not exhibit any cytotoxicity towards the tested cells. The results of the AFM investigation emphasized that the HGF-1 cells exhibited a good adherence to the CuHAp thin films’ surfaces, and there is also clear evidence of their spread all over the thin films’ surfaces even in the early stage of adhesion. The results of the AFM studies evidenced that the CuHAp thin films favored the adhesion and development of HGF-1 cells on their surface making them good candidates for future development of novel biocompatible devices for biomedical applications.

The results obtained by AFM investigation was correlated and confirmed the results of the MTT cell viability assay. More than that, the results obtained are in good concordance with other findings reported in the literature (Sahithi et al. 2010; Unabia et al. 2020; Bazin et al. 2021; Tuntun et al. 2023).

In their study regarding “sintering and biocompatibility of copper-doped hydroxyapatite bioceramics”, Bazin et al. (2021) have reported that copper-doped hydroxyapatite (CuHA) ceramics obtained using high temperature solid-state reaction sintering between HA and CuO powder mixtures ceramics do not cause sudden cell death in MC3T3-E1 cells. More than that, their study revealed that even after 5 days of incubation, the viability of the MC3T3-E1 cells were relatively high in all of the cases, therefore, emphasizing that copper ions do not exhibit a significant negative influence on the MC3T3-E1 cell’s physiology. Furthermore, their findings also determined that the cell viability was influenced by the copper concentration from the CuHA ceramics, showing that the highest decrease of cell viability was obtained for the CuHA0.7 sample, which contains the highest amount of copper. Nonetheless, the studies reported by Bazin et al. (2021) also determined that the presence of copper did manage to alter the cells functionality, and this behavior was attributed to the copper ability to generate of reactive oxygen species. Similar results were reported by Tuntun et al. (2023) in their study on the “crystallographic characterization and application of copper doped hydroxyapatite as a biomaterial”. Tuntun et al. reported that the 5% Cu doped HAp (CuHAp, with 5at%Cu) sample exhibited remarkable properties when their biocompatibility was investigated. Based on their results, they concluded that pure HAp as well as 5% and 10% Cu doped HAp can be considered in the development of implant materials.

## Conclusions

Hydroxyapatite (HAp) is used as a biocompatible and bioactive biomaterial in many applications in the biomedical field. The substitution of calcium ions with Cu ions led to the improvement of the properties of the thin films obtained by spin coating method. The surface properties of the CuHAp thin films were investigated by EDS, FTIR and XPS measurements. The qualitative chemical composition of the coatings was evaluated by EDS and XPS studies. The SEM results revealed that the CuHAp thin films surface is continuous and homogeneous. Moreover, the homogeneous repartition of the Ca, Cu, P and O in the studied sample was confirmed by the results of the elemental EDS cartographies studies. The results of the FTIR and Raman spectroscopy studies confirmed only the presence of the vibrational band that are specific to the hydroxyapatite structure in the studied CuHAp thin films. The results of the AFM studies revealed that the CuHAp thin layers exhibit a homogenous and continuous surface topography without any signs of cracks, fissures or other unevenness. The AFM studies showed that the CuHAp thin films enhanced the attachment and growth of HGF-1 cells on their surface, suggesting their potential for creating new biocompatible devices for biomedical purposes.
